# *Heracleum moellendorffii* roots inhibit the production of pro-inflammatory mediators through the inhibition of NF-κB and MAPK signaling, and activation of ROS/Nrf2/HO-1 signaling in LPS-stimulated RAW264.7 cells

**DOI:** 10.1186/s12906-019-2735-x

**Published:** 2019-11-12

**Authors:** Ha Na Kim, Jeong Dong Kim, Joo Ho Yeo, Ho-Jun Son, Su Bin Park, Gwang Hun Park, Hyun Ji Eo, Jin Boo Jeong

**Affiliations:** 1 Department of Medicinal Plant Resources, Andong National University, Andong, 36729 Republic of Korea; 2 Forest Medicinal Resources Research Center, National Institute of Forest Science, Yongju, 36040 Republic of Korea; 3 Insititute of Agricultural Science and Technology, Andong National University, Andong, 36729 Republic of Korea

**Keywords:** Anti-inflammation, *Heracleum moellendorffii*, Inflammatory diseases, Inflammatory response

## Abstract

**Background:**

*Heracleum moellendorffii* roots (HM-R) have been long treated for inflammatory diseases such as arthritis, backache and fever. However, an anti-inflammatory effect and the specific mechanism of HM-R were not yet clear. In this study, we for the first time explored the anti-inflammatory of HM-R.

**Methods:**

The cytotoxicity of HM-R against RAW264.7 cells was evaluated using MTT assay. The inhibition of NO and PGE_2_ production by HM-R was evaluated using Griess reagent and Prostaglandin E_2_ ELISA Kit, respectively. The changes in mRNA or protein level following HM-R treatment were assessed by RT-PCR and Western blot analysis, respectively.

**Results:**

HM-R dose-dependently blocked LPS-induced NO and PGE_2_ production. In addition, HM-R inhibited LPS-induced overexpression of iNOS, COX-2, IL-1β and IL-6 in RAW264.7 cells. HM-R inhibited LPS-induced NF-κB signaling activation through blocking IκB-α degradation and p65 nuclear accumulation. Furthermore, HM-R inhibited MAPK signaling activation by attenuating the phosphorylation of ERK1/2, p38 and JNK. HM-R increased nuclear accumulation of Nrf2 and HO-1 expression. However, NAC reduced the increased nuclear accumulation of Nrf2 and HO-1 expression by HM-R. In HPLC analysis, falcarinol was detected from HM-R as an anti-inflammatory compound.

**Conclusions:**

These results indicate that HM-R may exert anti-inflammatory activity by inhibiting NF-κB and MAPK signaling, and activating ROS/Nrf2/HO-1 signaling. These findings suggest that HM-R has a potential as a natural material for the development of anti-inflammatory drugs.

## Background

Macrophages, known as one of the immune cells, secrete a variety of pro-inflammatory mediators such as nitric oxide (NO), prostaglandin E_2_ (PGE_2_), inducible nitric oxide (iNOS) and cyclooxygenase-2 (COX-2), as well as pro-inflammatory cytokines including interleukin-1β (IL-1β), interleukin-6 (IL-6), and tumor necrosis factor-α (TNF-α) [1]. The appropriate cytokine secreted by macrophages acts to protect the body from external harmful factors, but excessive cytokines are known to cause chronic inflammation associated with inflammatory human diseases such as atheriosclerosis, arthritis, cardiovascular disease and other deadly diseases [2, 3]. Therefore, regulation of pro-inflammatory cytokines and mediators has been regarded as complementary strategy to the inflammatory human diseases.

*Heracleum moellendorffii* Hance (*H. moellendorffii*) growing in the field and mountains of Korea, China and Japan has been used as edible wild herb in Korea [4]. *H. moellendorffii* leaves have been reported to exert detoxification, antioxidant and anti-melanogenic activities [4–6] and *H. moellendorffii* roots have been used as traditional herbal medicine treating inflammatory human diseases such as arthritis, backache and fever [4]. In a previously reported study of the anti-inflammatory activity of *H. moellendorffii*, dehydrogeijerin isolated from *H. moellendorffii* leaves has been reported to block the expression of the pro-inflammatory mediators via the inhibition of MAPK signaling activation [7]. However, there is no studies on the anti-inflammatory activity and its potential mechanism of *H. moellendorffii* roots. In this study, we aimed to investigate anti-inflammatory activity of *H. moellendorffii* roots in LPS-stimulated RAW264.7 cells, and to elucidate the potential mechanism.

## Methods

### Materials

3-(4,5-dimethylthiazol-2-yl)-2,5-diphenyltetrazolium bromide (MTT), tolfenamic acid (TA), N-Acetylcysteine (NAC) and LPS were purchased from Sigma Aldrich (St. Louis, MO, USA). Antibodies against IκB-α, p65, phospho-ERK1/2, ERK1/2, phospho-p38, p38, phospho-JNK, JNK, HO-1, Nrf2, β-actin and TBP were purchased from Cell Signaling (Bervely, MA, USA).

### Sample preparation

After *H. moellendorffii* (voucher number: FMCHm-2019-0521-001~003) was collected and identified by Forest Medicinal Resources Research Center, National Institute of Forest Science (Yongju, Korea), *H. moellendorffii* was generously provided. Twenty gram of *H. moellendorffii* roots was immersed in 400 ml of 70% ethanol and then extracted for 72 h with stirring at room temperature. After 72 h, the extracts were filtered and concentrated using a vacuum evaporator and then lyophilized. The ethanol extracts of *H. moellendorffii* roots (HM-R) were stored − 80 °C until use. HM-R was dissolved in dimethyl sulfoxide (DMSO) before the experiment to treat the cells. DMSO was used as a control in all experiments and the concentration of DMSO treated in the cells did not exceed 0.1% (v/v).

### Cell culture

RAW264.7 cells (American Type Culture Collection, Manassas, VA, USA) were maintained at 37 °C under a humidified atmosphere of 5% CO_2_ using Modified Eagle medium (DMEM)/F-12 1:1 Modified medium (Lonza, Walkersville, MD, USA) containing 10% fetal bovine serum, 100 U/ml penicillin and 100 μg/ml streptomycin.

### Cell viability assay

The cytotoxicity of HM-R against RAW264.7 cells was evaluated using MTT assay. After the cells (3 × 10^3^ cells/well) were plated on a 96-well plate for 24 h, HM-R was applied to the cells for 24 h. Then, 50 μl of MTT solution (1 mg/ml) was added to the cells and incubated for 2 h. Then, cell culture supernatants were removed and DMSO was added to the cells. The absorbance was measured at 570 nm using UV/Visible spectrophotometer (Human Cop., Xma-3000PC, Seoul, Korea).

### NO and PGE_2_ determination

RAW264.7 cells (1 × 10^5^ cells/well) in 12-well plate for 24 h were pretreated with HM-R for 2 h and co-treated with LPS (1 μg/ml) for 18 h. After the treatment, the cell culture supernatants were collected for the analysis of NO and PGE_2_ production. For measurement of NO production, the cell culture supernatants and Griess reagent (Sigma Aldrich) were mixed at a 1:1 ratio and reacted at the room temperature for 15 min, and the absorbance was measured at 540 nm using UV/Visible spectrophotometer (Human Cop., Xma-3000PC, Seoul, Korea). PGE_2_ production was analyzed using Prostaglandin E_2_ ELISA Kit (Cayman Chemical, Ann Arbor, MI, USA) according to the manufacturer’s protocols.

### Isolation of nuclear fraction

After the treatment, nuclear protein from RAW264.7 cells was isolated using a Nuclear Extract Kit (Active Motif, Carlsbad, CA, USA) according to the manufacturer’s protocols. The isolated nuclear protein was stored at − 80 °C until analysis.

### SDS-PAGE and Western blot analysis

To extract protein from RAW264.7 cells, RAW264.7 cells were washed three times with cold 1 × phosphate-buffered saline and lysed at 4 °C for 30 min using cold radioimmunoprecipitation assay buffer (Boston Bio Products, Ashland, MA, USA) containing protease inhibitor (Sigma-Aldrich) and phosphatase inhibitor (Sigma-Aldrich). After centrifugation at 15,000 rpm for 10 min, the supernatant was recovered for protein quantitation using BCA protein assay (Thermo Fisher Scientific, Waltham, MA USA). The protein was separated on SDS-PAGE for about 1 h at 150 V and subsequently transferred to PVDF membrane (Bio-Rad Laboratories, Inc., Hercules, CA, USA) for 2 h at 100 V. After blocking the PVDF membranes using 5% non-fat dry milk in tris-buffered saline containing 0.05% Tween 20 (TBS-T) by stirring at room temperature for 1 h, the specific primary antibodies in 5% non-fat dry milk dissolved with TBS-T buffer were treated with PVDF membranes and reacted with stirring at 4 °C overnight. Then, PVDF membranes were washed three times with TBS-T buffer, and then treated with the secondary antibodies in 5% non-fat dry milk dissolved with TBS-T buffer for 1 h at room temperature. Chemiluminescence was detected with ECL Western blotting substrate (Amersham Biosciences, Piscataway, NJ, USA) and visualized using LI-COR C-DiGit Blot Scanner (Li-COR Biosciences, Lincoln, NE, USA).

### Reverse transcriptase-polymerase chain reaction (RT-PCR)

RNA isolation from RAW264.7 cells and cDNA synthesis from isolated RNA were performed using a RNeasy Mini Kit (Qiagen, Valencia, CA, USA) and a Verso cDNA Kit (Thermo Scientific, Pittsburgh, PA, USA) according to the manufacturer’s protocol, respectively. PCR was performed using PCR Master Mix Kit (Promega, Madison, WI, USA). The sequence of specific primers used for PCR analysis was as follows: iNOS: forward 5′-ttgtgcatcgacctaggctggaa-3′ and reverse 5′-gacctttcgcattagcatggaagc-3′, COX-2: forward 5′-gtactggctcatgctggacga-3′ and reverse 5′-caccatacactgccaggtcagcaa-3′, IL-1β: forward 5′-ggcaggcagtatcactcatt-3′ and reverse 5′-cccaaggccacaggtattt-3′, IL-6: forward 5′-gaggataccactcccaacagacc-3′ and reverse 5′-aagtgcatcatcgttgttcataca-3′; GAPDH: forward 5′-ggactgtggtcatgagcccttcca-3′ and reverse 5′-actcacggcaaattcaacggcac-3′. The PCR bands were visualized using agarose gel electrophoresis.

### NF-κB luciferase activity

Transient transfection was performed using the PolyJet DNA transfection reagent (SignaGen Laboratories, Ijamsville, MD, USA) according to the manufacturer’s protocol. Briefly, NF-κB luciferase construct (Addgene, Cambridge, MA, USA, 1 μg/well), pRL-null vector (0.1 μg/well) and PolyJet DNA transfection reagent were mixed for 15 min at room temperature. RAW264.7 cells were treated with the mixtures and incubated for 24 h. The measurement of NF-κB luciferase activity was performed using a dual-luciferase assay kit (Promega, Madison, WI, USA). pRL-null luciferase activity was used to normalize NF-κB luciferase activity.

### Analysis of bioactive components

The analysis of anti-inflammatory compounds from HM-R was performed using HPLC. In HPLC analysis, Waters 1525 system with a Waters 2487-dual λ absorbance detector was used. The column was equipped with the Waters SPHERISORB 10 μm Silica (250 mm × 4.6 mm). The mobile phase consisted of 10% ethanol and 90% hexane. The flow rate was kept constant at 1.0 ml/min for a total run time of 10 min. The injection volume of HM-R was 10 μl. The elution was monitored at 254 nm.

### Statistical analysis

All the data are shown as mean ± SD (standard deviation). Statistical analysis was performed with one-way ANOVA followed by Dunnett’s test. Differences with *P or #*P* < 0.05 were considered statistically significant.

## Results

### HM-R inhibits LPS-mediated overproduction of NO and PGE_2_ in RAW264.7 cells

**Fig. 1:**
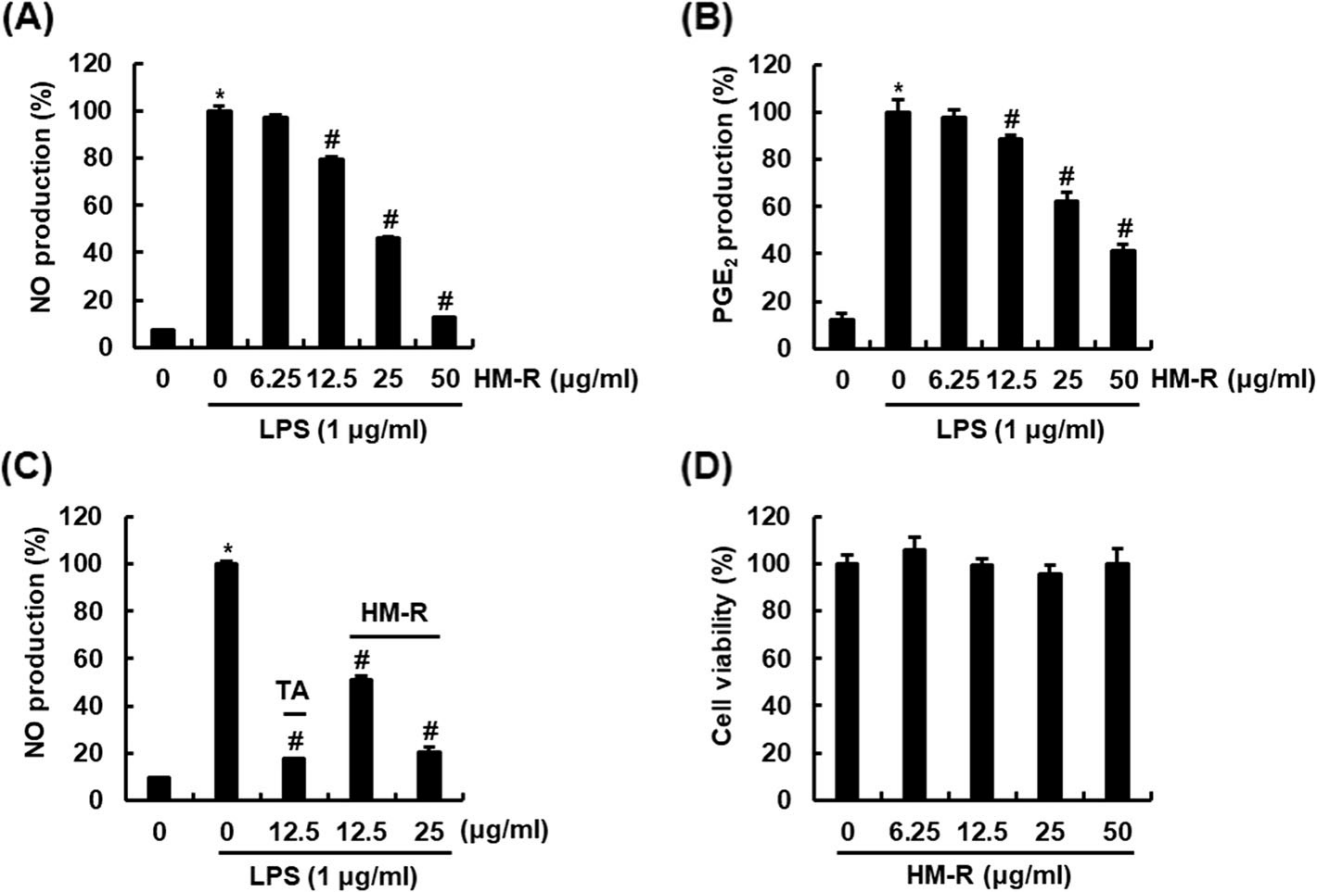
Inhibitory effect of HM-R against NO and PGE_2_ production in LPS-stimulated RAW264.7 cells. **a** and **b** RAW264.7 cells were pretreated with HM-R for 2 h and then co-treated with LPS (1 μg/ml) for 18 h. NO and PGE_2_ production was measured by Griess assay and Prostaglandin E_2_ ELISA Kit, respectively. **c** RAW264.7 cells were pretreated with HM-R or TA for 2 h and then co-treated with LPS (1 μg/ml) for 18 h. NO production was measured by Griess assay. **d** RAW264.7 cells were treated with HM-R for 24 h. Cell viability was measured by MTT assay. *P < 0.05 compared to the cells without the treatment, and ^#^*P* < 0.05 compared to the cells treated with LPS alone

HM-R reduced NO production by 20.3% at 12.5 μg/ml, 56.0% at 25 μg/ml and 88.4% at 50 μg/ml in LPS-stimulated RAW264.7 cells, respectively (Fig. 1a). It was also observed that HM-R inhibited LPS-induced overproduction of PGE_2_ by 11.3% at 12.5 μg/ml, 37.6% at 25 μg/ml and 58.7% at 50 μg/ml in RAW264.7 cells (Fig. 1b). We compared the inhibitory effect of HM-R against NO production with TA as one of the non-steroidal anti-inflammatory drugs (NSAIDs) in LPS-stimulated RAW264.7 cells. As shown in Fig. 1c, 25 μg/ml of HM-R showed similar inhibitory activity against LPS-mediated NO production compared to 12.5 μg/ml of TA. To investigate whether the inhibitory activity of HM-R was resulted from its cytotoxicity, the effects of HM-R on cell viability in RAW264.7 cells were measured using the MTT assay. HM-R did not affect cytotoxicity on RAW264.7 cells (Fig. 1d).

### HM-R inhibits LPS-mediated overexpression of pro-inflammatory mediators such as iNOS, COX-2, IL-1β and IL-6 in RAW264.7 cells

**Fig. 2:**
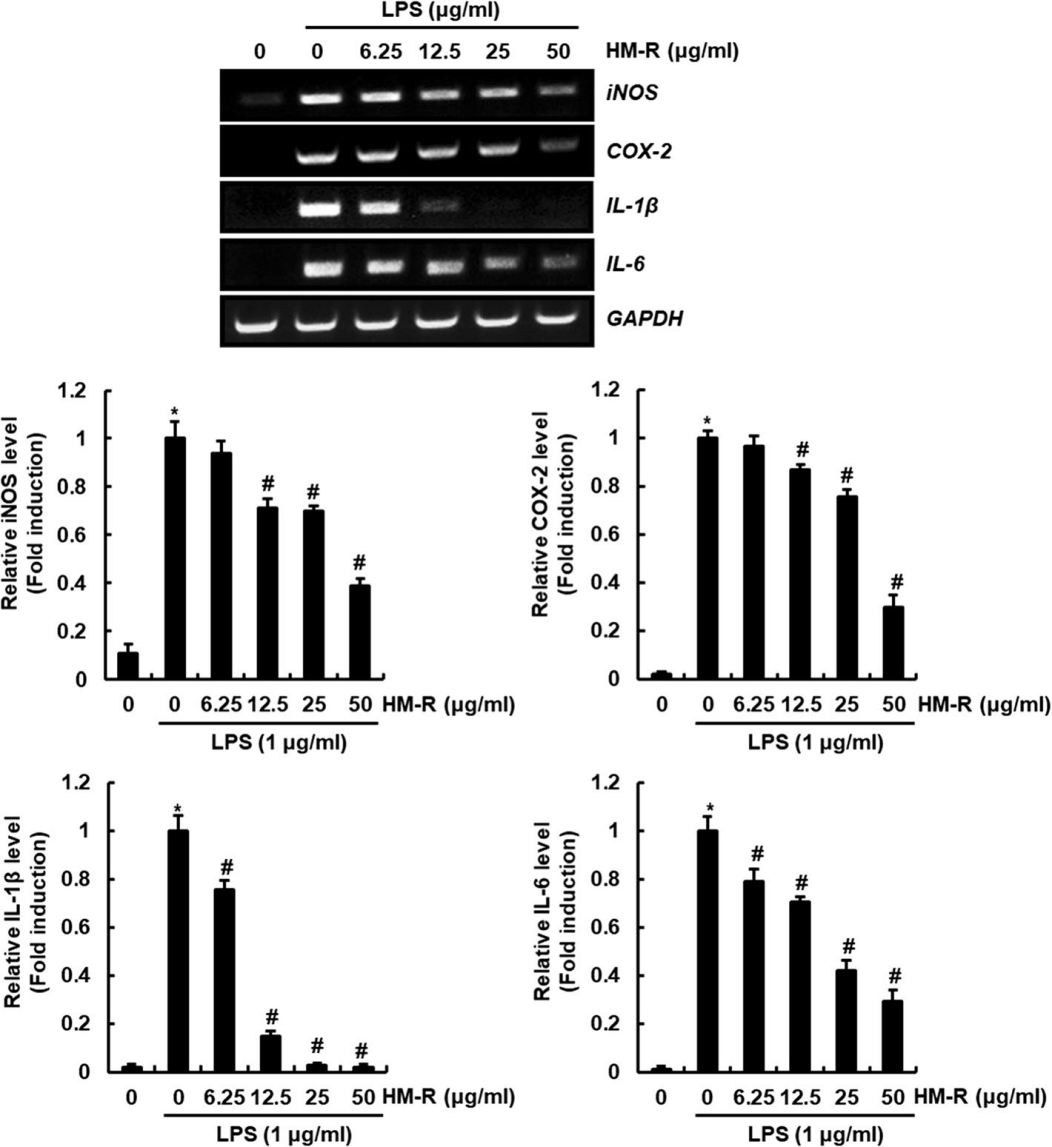
Inhibitory effect of HM-R against iNOS, COX-2, IL-1β and IL-6 in LPS-stimulated RAW264.7 cells. RAW264.7 cells were pretreated with HM-R for 2 h and then co-treated with LPS (1 μg/ml) for 18 h. Total RNA was prepared after LPS and HM-R treatment. GAPDH was used as internal control for RT-PCR. The density of mRNA bands was calculated using the software UN-SCAN-IT gel version 5.1 (Silk Scientific Inc. Orem, UT, USA). *P < 0.05 compared to the cells without the treatment, and ^*#*^*P* < 0.05 compared to the cells treated with LPS alone

In inflammatory response, the production of NO and PGE_2_ is regulated by the expression of iNOS and COX-2, respectively [8, 9]. Thus, we investigated the effect of HM-R on the expression of iNOS and COX-2 in LPS-stimulated RAW264.7 cells. As shown in Fig. 2, overexpression of iNOS and COX-2 was observed in the cells treated with LPS alone. However, HM-R has been shown to effectively inhibit the overexpression of iNOS and COX-2 induced by LPS. In addition, macrophages produce large amounts of inflammatory cytokines such as IL-1β and IL-6 that cause chronic inflammation when inflammatory response occurs [10]. Thus, we evaluated the inhibitory effect of HM-R against LPS-induced overexpression of IL-1β and IL-6. As a result (Fig. 2). HM-R dose-significantly suppressed the mRNA expression of IL-1β and IL-6 in LPS-stimulated RAW264.7 cells. Considering the inhibitory effect of HM-R on pro-inflammatory mediators, HM-R may be considered to have anti-inflammatory activity.

### HM-R inhibits LPS-mediated NF-κB and MAPK signaling activation in RAW264.7 cells

**Fig. 3:**
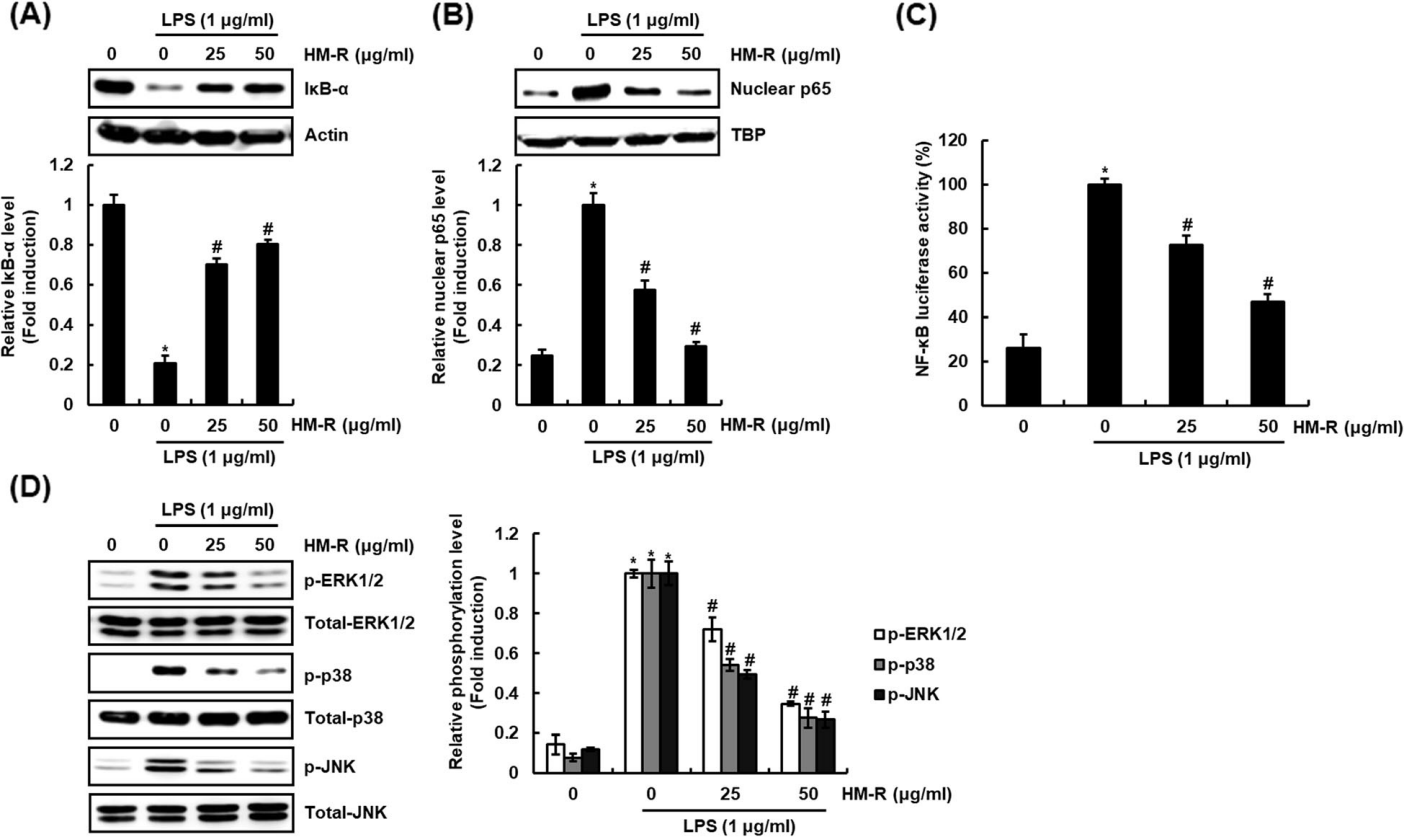
Inhibitory effect of HM-R against NF-κB and MAPK signaling activation in LPS-stimulated RAW264.7 cells. **a**, **d** RAW264.7 cells were pretreated with HM-R for 2 h and then co-treated with LPS (1 μg/ml) for 20 min. **b** RAW264.7 cells were pretreated with HM-R for 2 h and then co-treated with LPS (1 μg/ml) for 30 min. After the treatment, the nucleus fraction was prepared. For Western blot analysis, the cell lysates were subjected to SDS-PAGE and the Western blot was performed using antibodies against IκB-α and p65. Actin was used as internal control for Western blot analysis. The density of Western blot bands was calculated using the software UN-SCAN-IT gel version 5.1 (Silk Scientific Inc. Orem, UT, USA). *P < 0.05 compared to the cells without the treatment, and ^*#*^*P* < 0.05 compared to the cells treated with LPS alone. **c** RAW264.7 cells were co-transfected with NF-κB luciferase constructs and pRL-null. The cells were pretreated with HM-R for 2 h and then co-treated with LPS (1 μg/ml) for 18 h. Luciferase activity for NF-κB was measured as a ratio of firefly luciferase signal/renilla luciferase signal using a dual luciferase assay kit. *P < 0.05 compared to the cells without the treatment, and ^*#*^*P* < 0.05 compared to the cells treated with LPS alone

Abnormal activation of NF-κB and MAPK signaling in the inflammatory response results in excessive pro-inflammatory mediator production [11, 12]. Therefore, inhibition of NF-κB and MAPK signaling has been considered a major target for the development of anti-inflammatory drugs [13]. Thus, we investigated the inhibitory activity of HM-R against NF-κB and MAPK signaling to elucidate the mechanisms associated with anti-inflammatory activity of HM-R. Degradation of IκB-α by inflammatory stimuli such as LPS is essential for NF-κB signaling activation. As shown in Fig. 3a, the treatment of LPS alone induced IκB-α degradation, while the presence of HM-R significantly inhibited LPS-mediated degradation of IκB-α in RAW264.7 cells. Degradation of IκB-α results in nuclear accumulation of p65, and nuclear p65 binds to genes of pro-inflammatory mediators and induces expression of pro-inflammatory mediators [14]. Thus, we investigated the inhibitory activity of HM-R on p65 nuclear accumulation. As shown in Fig. 3b, HM-R inhibited LPS-induced nuclear accumulation of p65. In addition, HM-R dose-dependently suppressed LPS-induce activation of NF-κB luciferase activity (Fig. 3c).

In the regulation of MAPK signaling pathway by HM-R, the hyper-phosphorylation of ERK1/2, p38 and JNK was observed in the cells treated with LPS alone, but HM-R dose-dependently inhibited LPS-induced phosphorylation of ERK1/2, p38 and JNK. In view of these results, it is considered that inhibition of NF-κB and MAPK activation is a major signaling associated with the inhibition of HM-R against the expression of pro-inflammatory mediators.

### HM-R increases HO-1 expression through ROS-dependent Nrf2 activation in RAW264.7 cells

**Fig. 4:**
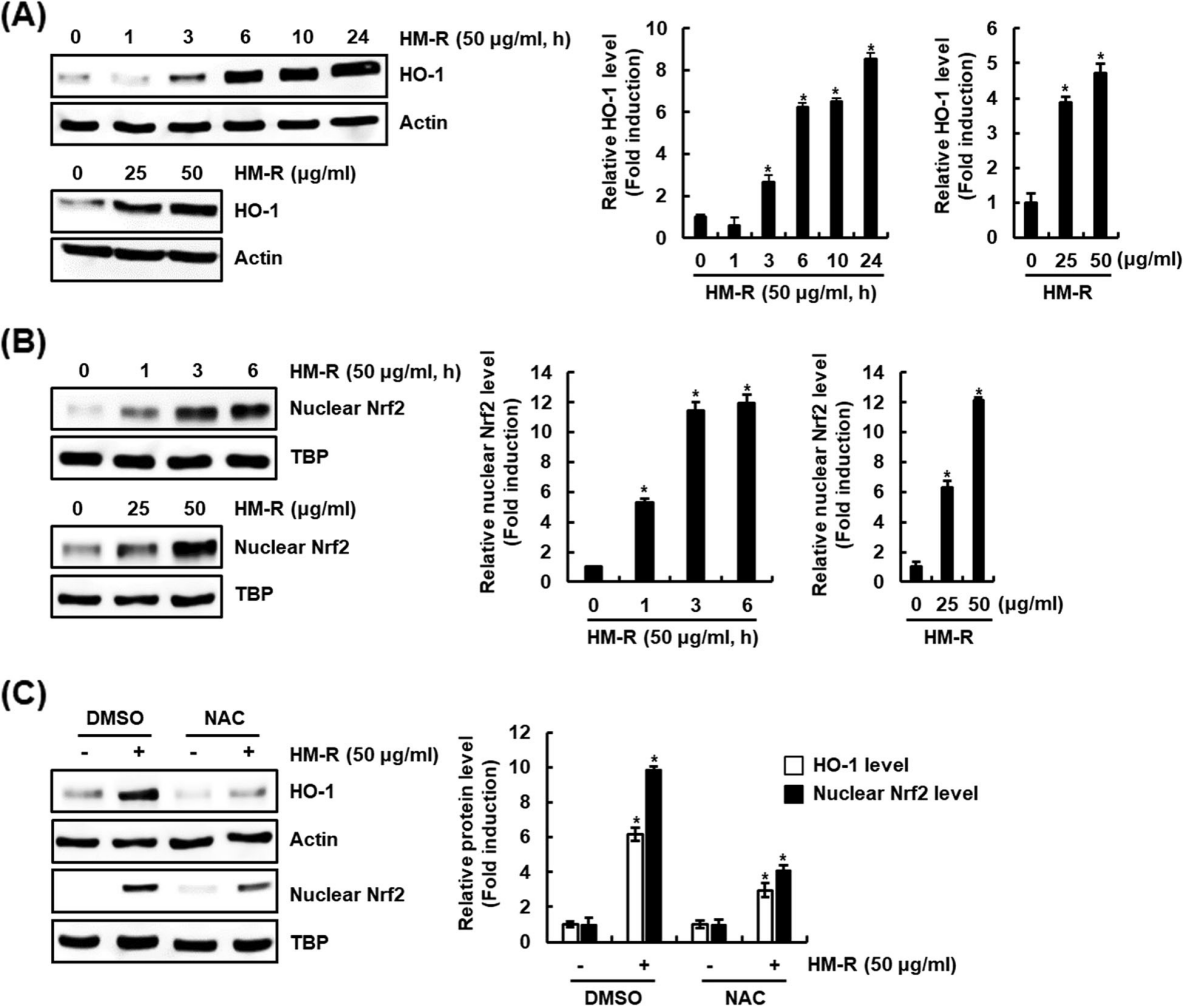
Effect of HM-R on HO-1 expression through ROS-dependent Nrf2 activation. **a** RAW264.7 cells were treated with HM-R (50 μg/ml) for the indicated times or indicated concentrations for 6 h. **b** RAW264.7 cells were treated with HM-R (50 μg/ml) for the indicated times or indicated concentrations for 3 h. After treatment, nuclear fraction was prepared. **c** RAW264.7 cells were pretreated with NAC (10 mM) for 2 h and then co-treated with HM-R for 6 h for HO-1 analysis or for 3 h for nuclear Nrf2 anaylsis. For Western blot analysis, the cell lysates were subjected to SDS-PAGE and the Western blot was performed using antibodies against HO-1 and Nrf2. Actin or TBP was used as internal control for Western blot analysis. The density of Western blot bands was calculated using the software UN-SCAN-IT gel version 5.1 (Silk Scientific Inc. Orem, UT, USA). *P < 0.05 compared to the cells without the treatment

Recently, Heme oxygenase-1 (HO-1) has been reported as an important molecular target for anti-inflammatory activity [15] and many natural products have been reported to exhibit anti-inflammatory activity through HO-1 expression dependent on activation of NF-E2-related factor 2 (Nrf2) [16–18]. Thus, we examined the effect of HM-R on HO-1 protein expression. As shown in Fig. 4a, HM-R increased the expression of HO-1 protein in a time and concentration-dependent manner. In addition, we observed that HM-R increased the nuclear accumulation of Nrf2 protein (Fig. 4b).

Reactive oxygen species (ROS) has been reported to increase expression of HO-1 protein through inducing nuclear accumulation of Nrf2 [19, 20]. In fact, Isoegomaketone as an essential oil component inhibited the production of pro-inflammatory mediators through ROS/Nrf2/HO-1 signaling activation [21]. Thus, the effect of ROS on Nrf2/HO-1 signaling activation by HM-R was investigated. As shown in Fig. 4c, the presence of NAC (ROS scavenger) blocked HM-R-mediated increase of HO-1 and nuclear Nrf2 level. These results suggest that activation of ROS/Nrf2/HO-1 signaling is anti-inflammatory signaling of HM-R.

### Analysis of bioactive components

**Fig. 5:**
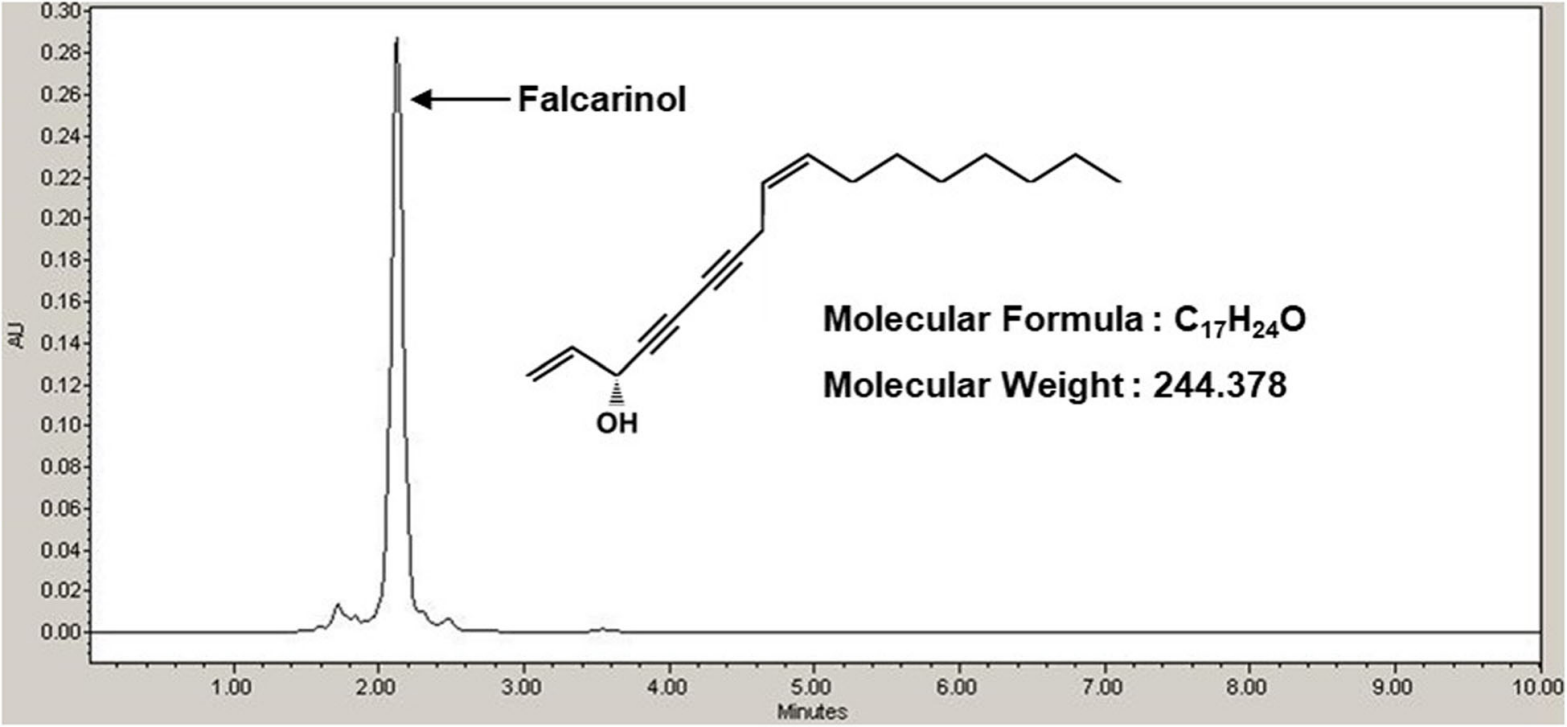
Chromatography of HPLC analysis of HM-R

To analyze the potential medicinal compounds with anti-inflammatory activity from HM-R, we performed HPLC analysis of HM-R. As shown in Fig. 5, HM-R was analyzed to contain falcarinol (Molecular formula: C_17_H_24_O, Molecular weight: 244.378). Indeed, HM-R was reported to have falcarinol [22]. In addition, falcarinol has been reported to exert anti-inflammatory activity [23].

## Discussion

Many synthetic drugs have been developed to treat inflammatory diseases, but long-term use of such synthetic drugs is known to cause a variety of side effects such as gastric ulcer, bleeding, cardiac abnormalities, bone marrow depression, renal dysfunction and bronchospasm in long-term use [24]. Thus, there is a need to develop more effective and safe anti-inflammatory drugs [25]. In this trend, medicinal plants which have been used for the treatment of inflammatory diseases in the past, have been considered important resources for the development of traditional knowledge-based anti-inflammatory drugs [25].

As traditional herbal medicine, *Heracleum moellendorffii* roots (HM-R) have traditionally been used to treat inflammatory human diseases such as arthritis, backache and fever [4]. However, the anti-inflammatory activity of HM-R has not been scientifically investigated. In order to develop anti-inflammatory drugs using traditional knowledge about the treatment of inflammatory diseases of HM-R, scientific evidence of HM-R’s anti-inflammatory activity and related mechanisms is need. Thus, we investigated anti-inflammatory activity and mechanism of action of HM-R in this study.

Although proper NO plays a major role in maintaining immunity and homeostasis, various human diseases related to inflammation are caused by excessive NO [26]. In addition, excessive PGE_2_ during inflammatory response is known to cause not only tissue damage, but also inflammatory diseases such as rheumatoid arthritis and chronic hepatitis [27]. In this study, we observed that HM-R blocked LPS-induced NO and PGE_2_ overproduction in RAW264.7 cells. Since NO and PGE_2_ are synthesized by iNOS and COX-2, respectively, the regulation of iNOS and COX-2 expression has been regarded to be important for suppression of excessive NO and PGE_2_ production [28]. Thus, the effect of HM-R on iNOS and COX-2 expression was investigated and we observed that HM-R inhibited LPS-mediated overexpression of iNOS and COX-2, which indicating that the inhibition of iNOS and COX-2 expression by HM-R may contribute to the attenuation of NO and PGE_2_ production. In addition, appropriate pro-inflammatory cytokines such as IL-1β and IL-6 contributes to the recovery of infection, but excessive accumulation of pro-inflammatory cytokines is known to cause chronic inflammation. Thus, the regulation of pro-inflammatory cytokines has been considered to be a complementary strategy for controlling the inflammatory disease process [2]. In this study, we observed that HM-R significantly inhibits IL-1β and IL-6 expression in LPS-stimulated RAW264.7 cells. These findings indicate that HM-R may exert anti-inflammatory activity. In order to confirm the degree of anti-inflammatory activity of HM-R, we compared the inhibitory effect of HM-R against LPS-induced overproduction of NO with tolfenamic acid (TA) as one of non-steroidal anti-inflammatory drugs. At the same concentrations (12.5 μg/ml) of HM-R and TA, HM-R showed lower inhibitory activity against LPS-induced NO production than TA, but 25 μg/ml of HM-R showed similar inhibitory activity compared to TA (12.5 μg/ml). Although HM-R had a lower inhibitory activity against LPS-induced NO production than TA, HM-R can be considered to be a potential source for the development of anti-inflammatory drugs because HM-R is a crude extract.

The elucidation of mechanism for pharmacological activity is important for the development of related drugs. LPS-induced inflammation is caused by inflammatory cascade signaling pathway, in which NF-κB has been known as a major transcription factor that regulates that production of pro-inflammatory mediators [29, 30]. Under inflammatory stimuli, NF-κB activation occurs through the phosphorylation and degradation of IκB-α, and subsequent p65 nuclear translocation. Nuclear p65 activates transcription of pro-inflammatory mediators [29, 30]. Consequently, HM-R blocked LPS-induced degradation of IκB-α and nuclear accumulation of p65, which resulted in the suppression of NF-κB activation. Similar to NF-κB signaling, LPS-activated MAPKs such as ERK1/2, p38 and JNK also play an important role in the generation of pro-inflammatory mediators [29, 31]. Furthermore, it has been known that MAPK is crucial for NF-κB activation and the binding of NF-κB to pro-inflammatory genes [32, 33]. In this study, HM-R significantly decreased the phosphorylation of ERK1/2, p38 and JNK. These findings indicate that HM-R may exert anti-inflammatory activity through the inhibition of NF-κB and MAPK signaling activation.

It is known that heme oxygenase-1 (HO-1), which catalyzes the degradation of heme into biliverdin, iron and carbon monoxide has anti-oxidant, anti-inflammatory and anti-proliferative functions [34, 35]. In fact, the anti-inflammatory activity of HO-1 has been demonstrated by various studies. It has been reported that overexpression of HO-1 prior to inflammatory stimulation inhibited expression of inflammatory mediators such as NO and IL-6 [36, 37]. In addition, severe inflammation appeared in mice model deficient in HO-1 [38]. These previous experimental evidence suggest that HO-1 may be a potential molecular target for treating inflammation [21]. NF-E2-related factor 2 (Nrf2), known as the upstream mediator of HO-1, is present in the cytoplasm under unstressed condition, while accumulated nuclear Nrf2 under the oxidative stress causes the expression of HO-1 [39]. In this study, we confirmed that nuclear accumulation of Nrf2 and HO-1 expression were increased in HM-R treated RAW264.7 cells. We also found that nuclear accumulation of Nrf2 and the increased expression of HO-1 by HM-R were reduced in NAC-treated RAW264.7 cells. These results indicate that HM-R may induce HO-1 expression through ROS-dependent Nrf2 activation, which contributes to anti-inflammatory activity.

In the analysis of anti-inflammatory compounds from HM-R using HPLC, falcarinol (Molecular formula: C_17_H_24_O, Molecular weight: 244.378) also known as panaxynol was analyzed. The previous study has reported that HM-R contains falcarinol [22]. In addition, falcarinol was reported to exert anti-inflammatory effect through Nrf2/HO-1 signaling activation [23].

## Conclusion

Taken together, these results show that HM-R inhibits the expression of pro-inflammatory mediators and cytokines through suppressing the NF-κB and MAPK signaling, and activating ROS/Nrf2/HO-1 signaling. These results can provide scientific evidence of traditional knowledge about the treatment of inflammatory diseases using HM-R. In addition, based on traditional knowledge, HM-R can be used as a resource for the development of anti-inflammatory drugs.

## Data Availability

The datasets used and/or analyzed during the current study available from the corresponding author on reasonable request.

## Abbreviations

COX-2: cyclooxygenase-2
HM-R: *Heracleum moellendorffii*
HO-1: Heme oxygenase-1
IL-1β: Interleukin-1β
IL-6: Interleukin-6
iNOS: Inducible nitric oxide synthase
LPS: Lipopolysaccharide
MAPK: Mitogen-activated protein kinase
MTT: 3-(4,5-dimethylthiazol-2-yl)-2,5-diphenyltetrazolium bromide
NF-κB: Nuclear factor-kappaB
NO: Nitric oxide
Nrf2: Nuclear factor erythroid 2-related factor 2
PGE_2_: prostaglandin E_2_
